# Biosynthetic Potential of a Novel Antarctic Actinobacterium *Marisediminicola antarctica* ZS314^T^ Revealed by Genomic Data Mining and Pigment Characterization

**DOI:** 10.3390/md17070388

**Published:** 2019-07-01

**Authors:** Li Liao, Shiyuan Su, Bin Zhao, Chengqi Fan, Jin Zhang, Huirong Li, Bo Chen

**Affiliations:** 1 SOA Key Laboratory for Polar Science, Polar Research Institute of China, 451 Jinqiao Road, Shanghai 200136, China; 2 College of Marine Sciences, Shanghai Ocean University, Shanghai 201306, China; 3 School of Biotechnology, East China University of Science and Technology, Shanghai 200237, China; 4 Key Laboratory of East China Sea & Oceanic Fishery Resources Exploitation and Utilization, Ministry of Agriculture, East China Sea Fisheries Research Institute, Chinese Academy of Fishery Sciences, Shanghai 200090, China

**Keywords:** *Marisediminicola*, Antarctica, carotenoid, actinobacteria, natural products, gene cluster

## Abstract

Rare actinobacterial species are considered as potential resources of new natural products. *Marisediminicola antarctica* ZS314^T^ is the only type strain of the novel actinobacterial genus *Marisediminicola* isolated from intertidal sediments in East Antarctica. The strain ZS314^T^ was able to produce reddish orange pigments at low temperatures, showing characteristics of carotenoids. To understand the biosynthetic potential of this strain, the genome was completely sequenced for data mining. The complete genome had 3,352,609 base pairs (bp), much smaller than most genomes of actinomycetes. Five biosynthetic gene clusters (BGCs) were predicted in the genome, including a gene cluster responsible for the biosynthesis of C50 carotenoid, and four additional BGCs of unknown oligosaccharide, salinixanthin, alkylresorcinol derivatives, and NRPS (non-ribosomal peptide synthetase) or amino acid-derived compounds. Further experimental characterization indicated that the strain may produce C.p.450-like carotenoids, supporting the genomic data analysis. A new xanthorhodopsin gene was discovered along with the analysis of the salinixanthin biosynthetic gene cluster. Since little is known about this genus, this work improves our understanding of its biosynthetic potential and provides opportunities for further investigation of natural products and strategies for adaptation to the extreme Antarctic environment.

## 1. Introduction

Actinobacteria are considered as one of the most important producers of natural products for drug discovery. To date, more than half of antibiotics were produced by the genus *Streptomyces* [1]. However, the re-discovery of known natural products, especially from the genus *Streptomyces* due to the so-called diminishing marginal effect, has become an issue [1,2]. Meanwhile, the increased prevalence of multi-drug resistance pathogens and the emergence of cross-resistance to antibiotics have driven researchers to seek for novel natural products [3]. Therefore, rare actinobacteria (non-*Streptomyces*), especially those from underexplored extreme environments, draw increasing attention for discovering novel bioactive compounds [4]. Antarctica, a frozen continent surrounded by the Southern Ocean, is characterized by extremely low temperatures, strong ultraviolet radiation and a relatively isolated ecosystem [5]. It represents one of the most under-investigated environments. Hence, it was considered as a huge reservoir of microorganisms with biosynthetic potential of novel natural products. 

The genus *Marisediminicola* belonging to rare actinobacteria was first proposed nine years ago [6], with the only type species *Marisediminicola antarctica* and the only type strain *M. antarctica* ZS314^T^. The strain ZS314^T^ was isolated from intertidal sediments of the coast off the Chinese Antarctic Zhongshan Station in East Antarctica (69°22′13″ S, 76°21′41″ E). To date, no other type strains have been reported belonging to this species or this genus. However, many 16S rRNA sequences sharing over 99% identities have been deposited in the NCBI GenBank database, with most of them from the Arctic, Antarctica or glacier-associated environments. It suggests that *M. antarctica* is a phylotype adapted and probably specific to extreme environments. The strain ZS314^T^ was psychrophilic, with the growth temperature range of 0–26 °C (optimum 18–23°C). It developed a bright reddish orange color, which is usually caused by various carotenoid pigments. Carotenoids are a group of natural isoprenoid pigments mainly isolated from a wide variety of plants, algae, fungi and bacteria [7,8,9,10,11,12]. The major functions of known carotenoids are to prevent cells from oxidative damage [8], protect against UV radiation and modulate membrane fluidity in bacteria [7]. Therefore, carotenoids are important not only for the survival of the organisms but also for industrial applications.

To investigate the biosynthetic abilities of *M. antarctica* ZS314^T^, we performed genome sequencing and bioinformatics data mining. Further experiments including HPLC/TOF-MS (high-performance liquid chromatography/time-of-flight mass spectrometry) and anti-oxidation assays were carried out to characterize the pigments, as supported by the presence of a carotenoid biosynthetic gene cluster and the indicative color. *M. antarctica* was identified and reported nine years ago, but still little is known about this novel genus/species. Here, we report the findings through wet lab experiments and data mining of the genome to increase our understanding of this novel Antarctic actinobacterium. This study represents the first investigation of the novel Antarctic *Marisediminicola* genus for its potential in natural product biosynthesis, and also the first complete genome of the genus for future studies. 

## 2. Results and Discussion

### 2.1. General Feature of the Genome

The complete genome of *M. antarctica* ZS314^T^ was circular, containing 3,352,609 bp with an average GC content of 67.17% (Figure S1). Compared with other actinobacteria, *M. antarctica* species tended to have a relatively small genome (3.35 Mb vs. the largest actinobacterial genome of 13.05 Mb from *Nonomuraea* sp. ATCC 55076) and low GC content (67.17% vs. the highest GC content of 74.8 % in *Streptomyces*). To get an idea of the average genome size of the phylum Actinobacteria, a total of 1674 genomes (complete genomes and genomes assembled to the chromosome level) available in the GenBank database were analyzed. The resulting average genome size was 4.52 Mb. Meanwhile, the average GC content was 64.12% and the average number of CDSs was around 3937. Therefore, *M. antarctica* ZS314^T^ has a genome size below average but a GC content slightly higher than average. A total of 2992 (below average) protein-encoding genes were annotated in the genome, with up to 36% genes annotated as hypothetical proteins or without significant similar matches in the NCBI nr (non-redundant) database. Up to 44%, 41%, 36%, and 23% of genes had no match in the Swiss-Prot, gene ontology (GO), Kyoto encyclopedia of genes and genomes (KEGG) and clusters of orthologous groups (COG) databases, respectively. Two *rrn* operons and 44 tRNA genes were detected in the genome. The complete genome can be further analyzed for genome evolution and genetic adaptation to the extreme environment. 

### 2.2. Prediction of Secondary Metabolite Biosynthetic Gene Clusters

**Figure 1:**
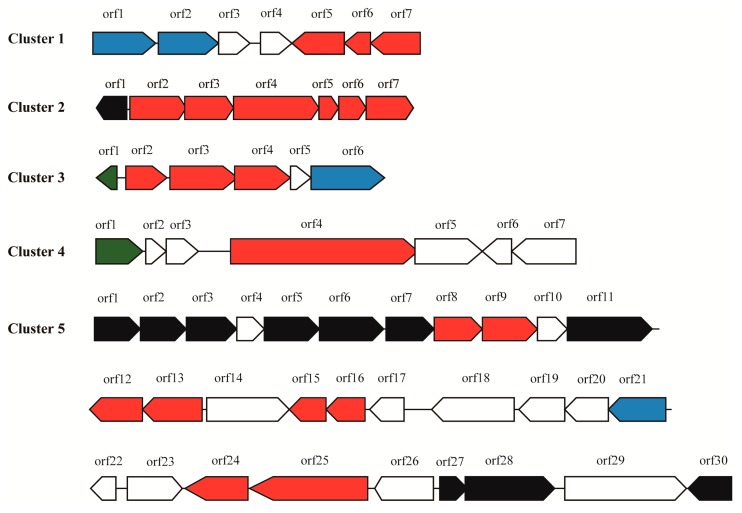
The five gene clusters predicted in the genome of *M. antarctica* ZS314^T^. Core biosynthetic genes are indicated in red, additional biosynthetic genes in black, transporter genes in blue, regulator genes in green, and hypothetical protein-encoding genes or unrelated genes in white.

Five secondary metabolite BGCs were predicted by the antiSMASH software (Table 1 and Table S1, and Figure 1). Since most BGCs are large (over 20 kb to around 100 kb), more BGCs are usually found in larger genomes (e.g., about 20 to 40 BGCs are generally predicted in *Streptomyces* genomes over 9 Mb). Although fewer BGCs were found in the genome of *M. antarctica* ZS314^T^ compared with *Streptomyces*, the difference of relative abundance of BGCs normalized to genome size is small. In the relatively small genome of *M. antarctica* ZS314^T^, the presence of the five gene clusters suggests their importance to the strain. 

#### 2.2.1. Cluster 1: T3PKS

After manual analysis, cluster 1 (Figure 1) was found to contain three core biosynthetic genes encoding stilbene synthase (*srs*A, orf7), isoprenylcysteine carboxyl methyltransferase (*srs*B, orf6) and monooxygenase (*srsC*, orf5), sharing 51–63% amino acid identities with the *srs* operon of *Streptomyces griseus* NBRC 13350 [13]. Two putative transporter-encoding genes (orf1 and orf2) were detected in the opposite orientation upstream of orf3 and orf4, encoding a hypothetical protein and a haloacid dehalogenase. The two genes (orf3 and orf4) seem not to be related to the *srs* operon since the three core genes (*srs*ABC) alone can biosynthesize alkylresorcinols. SrsA is a specialized type III polyketide synthase (PKS), catalyzing the formation of alkylresorcinols from acyl-CoAs of various chain lengths [13,14]. The alkylresorcinols are further modified by SrsB and SrsC through methylation and hydroxylation [13]. SrsA plays a key role in determining the structure of alkylresorcinols. The most similar record of SrsA from *M. antarctica* ZS314^T^ in the NCBI nr database was a stilbene synthase from *Agreia* sp. leaf244, sharing 65% amino acid identity. However, SrsA from *M. antarctica* ZS314^T^ was separated from the known stilbene synthases including the most similar one, as shown in the phylogenetic tree of related stilbene synthases found in the NCBI nr database (Figure S2). It suggests that this specialized type III PKS is distinct from the known enzymes, and hence indicates the production of alkyresorcinols with potential new chemical structure. Alkyresorcinols, a type of non-isoprenoid lipids with an aromatic ring, have important antibiotic activities [13,14]. Alkyresorcinols are relatively rare in nature, with plants as the major sources. Some species of bacteria are known to produce alkyresorcinols, such as *Streptomyces griseus* [13], *Azotobacter* and *Pseudomonas* species [15]. The detection of this putative alkyresorcinol biosynthetic gene cluster extends its distribution to this novel genus.

#### 2.2.2. Cluster 2: Terpene

**Figure 2:**
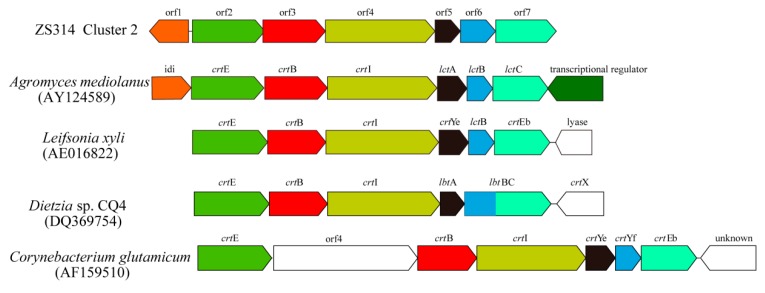
The C_50_ carotenoid synthesis gene clusters of *M. antarctica* ZS314^T^, *Dietzia* sp. CQ4, *Corynebacterium glutamicum*, *Leifsonia xyli* and *Agromyces mediolanus*. Homologous genes are presented in the same colors. Genes unrelated to the biosynthesis are denoted in white.

Cluster 2 was predicted to produce terpene type compounds, a large and diverse group of natural products [2]. Cluster 2 shared some similarity in structure and sequence with four C_50_ carotenoid synthetic gene clusters from *Dietzia* sp. CQ4, *Corynebacterium glutamicum*, *Leifsonia xyli* and *Agromyces mediolanus* (Figure 2 and Table 2). The three genes (orf2 to orf4) in cluster 2 of *M. antarctica* ZS314^T^ shared 34–61% amino acid identities with geranylgeranyl pyrophosphate synthase gene (*crt*E), phytoene synthase gene (*crt*B) and phytoene desaturase gene (*crt*I) of the four gene clusters, respectively (Table 2). The three genes *crt*EBI were reported to be core genes responsible for biosynthesis of C_40_ carotenoid lycopene. In addition, two genes (orf5 and orf6) encoding C_50_ carotenoid cyclases and orf7 encoding a lycopene elongase were predicted in cluster 2, sharing 35–65% amino acid identities with those in the other four gene clusters (Table 2). These three genes (orf5 to orf7) were expected to convert C_40_ carotenoid lycopene to C_50_ carotenoids, by elongating the carbon chain and forming cyclized end groups finally, as reported in previous studies [9,12]. In certain instances, one C_50_ carotenoid cyclase encoding gene could be fused with the lycopene elongase gene, such as *lbt*BC in *Dietzia* sp. CQ4 (Figure 2). A few additional genes were present in some of the clusters. For example, both cluster 2 of *M. antarctica* ZS314^T^ and the cluster from *Agromyces mediolanus* contained a gene (orf1/*idi*) encoding isopentenyl-diphosphate Delta-isomerase, which is responsible for isoprenoid biosynthesis. The cluster of *Dietzia* sp. CQ4 contained *crt*X encoding a glycosyl transferase, likely involved in the glucosylation of C_50_ carotenoids [12]. In addition, a transcriptional regulator was encoded with opposite orientation in the gene cluster of *Agromyces mediolanus*. Similarly, a putative transcriptional regulator of MarR family was detected close to the core biosynthetic genes in cluster 2 with opposite orientation. 

**Figure 3:**
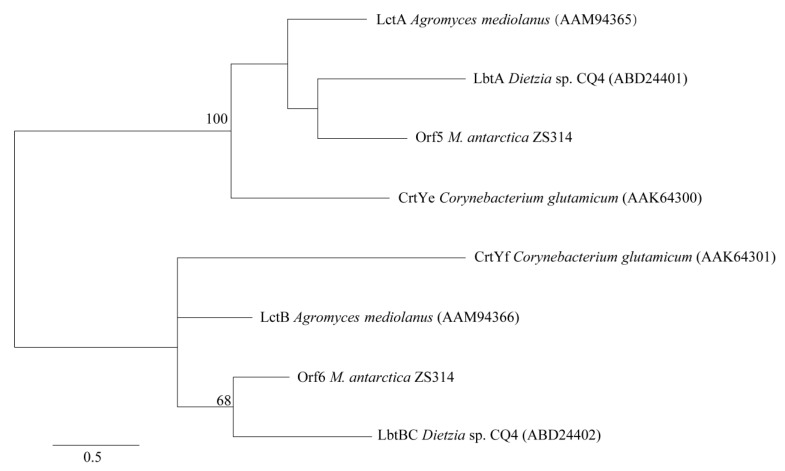
Maximum likelihood phylogenetic tree of C_50_ carotenoid cyclases based on amino acid sequences. Bootstrap values below 50 were not shown.

Although the gene clusters responsible for C_50_ carotenoids were quite similar, the final products had different structures. The final product of the gene cluster in *Dietzia* sp. CQ4 was C_50_ β-cyclic carotenoid (C.p.450 monoglucoside) [12], while the final products of the two clusters in *Corynebacterium glutamicum* and *Agromyces mediolanus* were the same, i.e., C_50_ ε-cyclic carotenoid (decaprenoxanthin) [12,16]. The final product of the gene cluster in *Leifsonia xyli* was predicted to be a putative C_50_ carotenoid, without further identification [12]. As previously reported, the C_50_ carotenoid cyclase was the key to determine the final structure and may have multiple catalytic functions [9]. Usually, the C_50_ carotenoid cyclase in Gram-positive bacteria was reported to be encoded by two genes, forming a heterodimeric complex to catalyze the cyclization reaction leading to carotenoids with different end groups [9,17]. Phylogenetic analysis of the C_50_ carotenoid cyclases showed that the two monomers formed two different groups in the tree (Figure 3). The C_50_ carotenoid cyclases (encoded by orf5 an orf6) of *M. antarctica* ZS314^T^ clustered with LbtA and LbtBC of *Dietzia* sp. CQ4, respectively (Figure 3). It suggests that cluster 2 of *M. antarctica* ZS314^T^ may produce C_50_ β-cyclic carotenoid-like products, as in *Dietzia* sp. CQ4. However, it is hard to predict the final products of the gene cluster 2 of *M. antarctica* ZS314^T^ solely based on the gene cluster analysis.

#### 2.2.3. Cluster 3: Terpene

**Figure 4:**
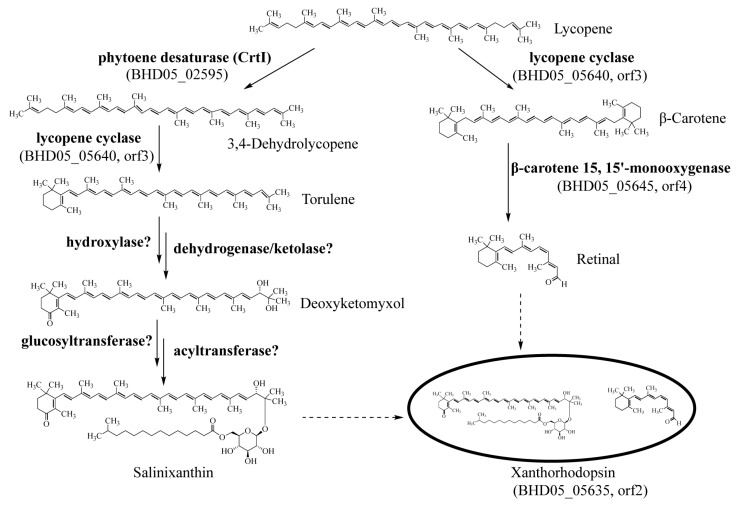
Proposed pathways for the biosynthesis of the two chromophores, salinixanthin and retinal, of xanthorhodopsin from *M. antarctica* ZS314^T^.

Cluster 3 was predicted to be a terpene-producing gene cluster due to the presence of a core biosynthetic gene (orf3) encoding lycopene cyclase (Figure 1 and Table S1). Next to the orf3 was a gene encoding xanthorhodopsin (orf2). Xanthorhodopsin is a light-driven proton pump with a dual chromophore, containing one retinal and one salinixanthin [18,19]. Xanthorhodopsin is more effective than bacteriorhodopsin for collecting light due to the presence of salinixanthin. Salinixanthin is a C-40 acyl glycoside carotenoid, a kind of rare carotenoid produced mostly from haloarchaea and halophilic bacteria [20]. However, the biosynthesis of salinixanthin has not been reported yet, according to our knowledge. A gene annotated as β-carotene 15,15’-monooxygenase (orf4) was found downstream of the core biosynthetic gene (orf3), which was reported to catalyze the breakdown of β-carotene into two molecules of retinal. A putative pathway for salinixanthin and retinal biosynthesis was hence proposed (Figure 4), according to reactions in the KEGG database and the genes existed in cluster 3 and the genome. Lycopene, which can be biosynthesized by the three genes *crt*EBI in cluster 2, was further converted to 3,4-dehydrolycopene by CtrI. The later was cyclized at one end to form torulene by most likely the lycopene cyclase in cluster 3. The reactions from lycopene to torulene were the same as the steps in the biosynthesis of neurosporaxanthin [21]. From torulene to salinixanthin, hydroxylase, dehydrogenase or ketolase, glucosyltransferase, and acyltransferase were proposed to catalyze the reactions, according to similar reactions in the biosynthesis of canthaxanthin [22] and myxol [23] (Figure 4). Gene candidates encoding for these enzymes have been annotated in the genome, however, not in cluster 3. We also performed metabolic pathway reconstruction for the biosynthesis of salinixanthin using PathPred, a web-based server integrated into the KEGG database to predict plausible enzyme-catalyzed reaction pathways using the information of biochemical structure transformation patterns and chemical structure alignments of substrate-product pairs [24]. The reconstructed pathway from lycopene to the core structure of salinixanthin without glucoside ester modification was shown in Figure S3 (since pathway reconstruction from lycopene to salinixanthin was frequently disrupted due to some unknown reason). The predicted pathway was quite complex, involving up to 19 reactions different from the pathway we proposed. It is unknown whether both pathways are possible without further experimental verification. Genetic manipulation and chemical identification are encouraged to verify the hypothetical pathways and identify the enzymes responsible for the catalysis of these reactions. Moreover, genome-scale metabolic pathway reconstruction can be used to further discover new metabolic pathways and analyze metabolic networks of *M. antarctica* ZS314^T^, as shown in previous studies [25,26].

**Figure 5:**
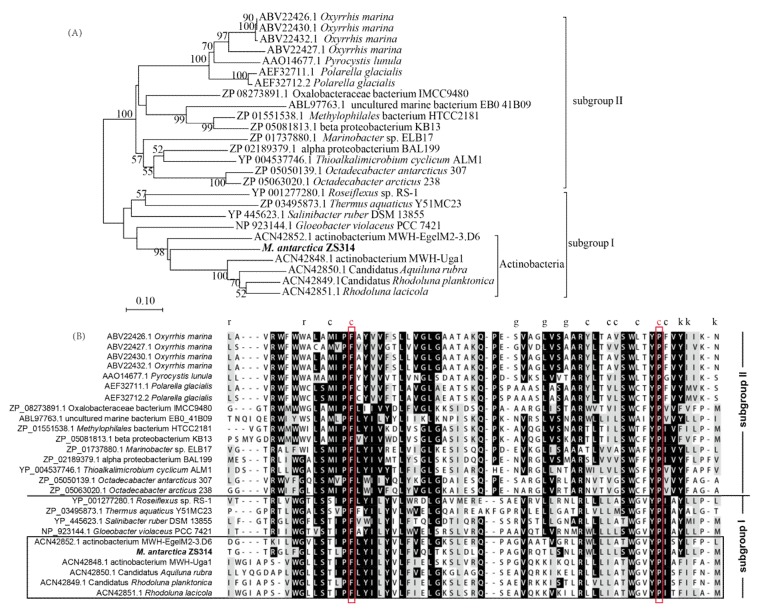
Comparison of xanthorhodopsins from *M. antarctica* ZS314^T^ and other representative microbial groups used in a previous study [27]. (**A**) Neighbor-joining tree of xanthorhodopsins based on amino acid sequences. Bootstrap values below 50 were not shown. (**B**) Multiple alignments of the putative keto-carotenoid-binding region of the xanthorhodopsins in (**A**), as identified previously [27,28]. Same as in the previous study [27], residues are marked by the letters r, c, g, and k to indicate contact with the ring, chain, glucoside and keto group of the carotenoid. The alignment was made using the ClustalW program online (https://www.genome.jp/tools-bin/clustalw) and shaded by the BoxShade program (https://embnet.vital-it.ch/software/BOX_form.html). Similar and identical residues in the alignment are highlighted with grey and black colors on the background, respectively.

Xanthorhodopsin was first discovered in *Salinibacter ruber*, a halophilic bacterium [18]. Later, two *Octadecabacter* strains from the Antarctic and the Arctic were reported to have a new subgroup of xanthorhophosins and were predicted to be an adaptation advantage to icy environments [27]. The xanthorhodopsin from *M. antarctica* ZS314^T^ clustered with those from Actinobacteria within the subgroup I as proposed previously (Figure 5) [27]. However, it was separated from the known xanthorhodopsin genes within the subgroup I with the highest similarity of around 57%, indicating that it was a new xanthorhodopsin gene. Although xanthorhodopsin genes were horizontally transferred, it seems that Actinobacteria shared a common ancestor distinct from non-actinobacterial genes. The residues involved in keto-carotenoid binding identified in *Salinibacter ruber* DSM 13855 were not conserved, except for two residues highlighted in red boxes (Figure 5b). It suggests that xanthorhodopsins may adopt different mechanisms for chromophore binding. 

#### 2.2.4. Cluster 4: NRPS

A putative cluster containing an NRPS gene as the core biosynthetic gene (orf4) was predicted right next to cluster 3 (Figure 1 and Table S1). However, the NRPS only contained an adenylation (A) domain, a peptidyl carrier protein (PCP) domain, and a termination (T) domain, lacking the required condensation (C) domain for elongation. In addition, the NRPS gene is much smaller than those multimodular assembly lines for non-ribosomal peptide compound biosynthesis. A peptidase M1 gene (orf5) followed the NRPS gene. No other biosynthetic gene was detected in the cluster (Figure 1). Therefore, it seems that cluster 4 may not produce large non-ribosomal peptide compounds or may produce single amino acid-derived compounds. However, homologs of this NRPS gene were found in other bacteria including mostly *Cryobacterium* species within Microbacteriaceae of Actinobacteria, with 76.86% as the highest amino acid identity. It is unclear whether this NRPS gene is inactivated or not. The function of this NRPS gene requires further investigation.

#### 2.2.5. Cluster 5: Oligosaccharide

Cluster 5 was predicted to biosynthesize oligosaccharides, including genes encoding polysaccharide biosynthesis protein, glycosyltransferases and epimerases etc. (Figure 1 and Table S1). However, no known gene cluster was found to be similar. Currently, only a few gene clusters biosynthesizing oligosaccharides have been identified, such as gene clusters producing orthosomycins, moenomycins, saccharomucins, and acarviostatins [29]. In general, these gene clusters are complex and large, usually including multiple genes for sugar synthesis and modification. For example, there are 39 biosynthetic related genes in the gene cluster producing avilamycin (a member of orthosomycins), including four glycosyltransferases, 22 sugar synthesis and tailoring genes, and other genes involved in further modification, resistance, regulation, and transport [30]. Due to the complexity of oligosaccharide producing gene clusters, the biosynthesis of oligosaccharides is lagged behind. Indeed, oligosaccharide natural products are under-represented in pharmacopeia compared with other classes, although oligosaccharides have a wide range of biological activities and high structural diversity [29]. Possible reasons were proposed including low levels of expression in nature, difficulties in detecting, isolation, and identification, as well as low stability [29]. Since oligosaccharide secondary metabolite BGCs are rarely identified and biochemically characterized, the prediction of these gene clusters in genomes is also less frequent than other classes of natural products. Therefore, further identification of the putative oligosaccharide gene cluster 5 is important but challengeable.

### 2.3. Analysis of the Pigments from M. antarctica ZS314^T^

**Figure 6:**
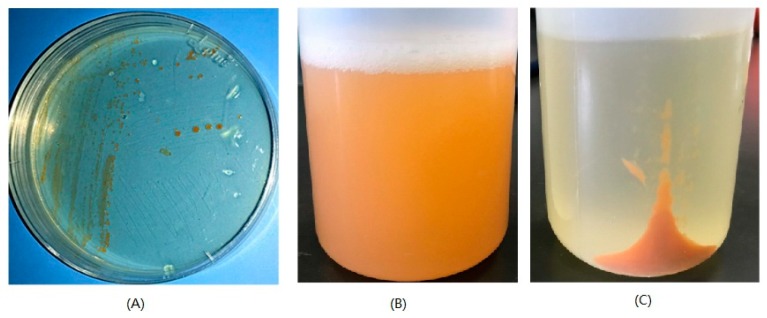
Reddish orange pigments produced by *M. antarctica* ZS314^T^ as observed on Marine Agar 2216 plate (**A**) and in Marine Broth 2216 before (**B**) and after (**C**) centrifugation.

**Figure 7:**
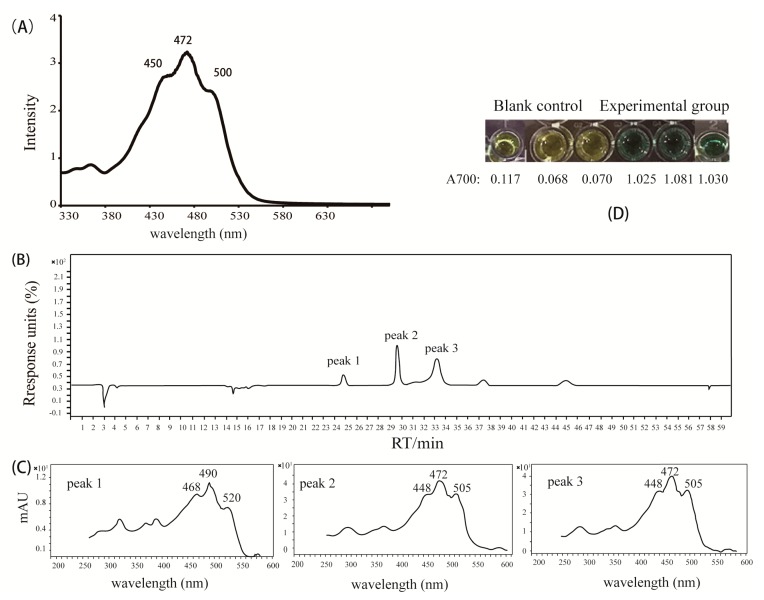
Analysis of the concentrated methanolic extracts of *M. antarctica* ZS314^T^. (**A**) UV-visible absorption spectra of total methanolic extracts. (**B**) HPLC chromatogram of the concentrated methanolic extracts showing three peaks (1, 2, 3) with typical UV absorption spectra of carotenoids. (**C**) The UV-visible absorption spectra of peaks 1, 2, and 3. (**D**) The anti-oxidation assay (FRAP assay) of total methanolic extracts.

As revealed by the genomic data mining, a putative carotenoid biosynthetic gene cluster was detected, suggesting the production of carotenoids. The physiological characteristics indicated the production of carotenoid-like pigments as well. Colonies of *M. antarctica* ZS314^T^ were reddish orange on Marine Agar 2216 (Difco) (Figure 6A). The supernatant of liquid culture was almost colorless, and the cell pellets were reddish orange after centrifugation (Figure 6B,C). It suggested that the pigments were stored in the cells rather than being secreted into the broth. The UV-visible absorption spectrum of the concentrated methanolic extracts showed three major absorption peaks at 450, 472, and 500 nm, with the highest at 472 nm (Figure 7A). The shape of the absorption spectrum resembled that of carotenoids in general, and the wavelengths fell within the range of carotenoids as well, indicating the presence of carotenoids in the extracts. The concentrated methanolic extracts were separated by using HPLC, resulting in three major peaks (peak 1, peak 2 and peak 3) at retention times of 24.786 min, 29.661 min and 33.284 min (Figure 7B). The three peaks showed UV-visible absorption spectrum characteristic of carotenoids, indicating that carotenoids may exist in the peaks. The absorption spectra of the three peaks were slightly different from the overall spectrum of total methanolic extracts. Peak 1 showed maximum absorptions at around 468 nm, 490 nm, and 520 nm (Figure 7C). Peaks 2 and 3 had similar absorption spectra, with three maximum absorptions at around 448 nm, 472 nm, and 505 nm (Figure 7C). In order to isolate putative carotenoids indicated by HPLC, three pooled fractions showing indicative color and relatively good purity were obtained after column chromatography with silica gel and Sephadex LH-20. Fraction 1 had one peak showing characteristic absorption spectrum of carotenoids (λ_max_ 452 and 480) (data not shown), with the major MS signal MH^+^ at 704.54898 (Figure S4), which is close to the molecular mass of C.p.450, sarcinaxanthin and flavuxanthin [9,12]. In addition, the same absorption spectrum was reported for C.p.450, rather than the other two carotenoids. Therefore, the preliminary data suggested that fraction 1 was likely C.p.450. Fraction 2 had one putative carotenoid-like peak (λ_max_ 450 and 475) (data not shown), showing the major MS signal MH^+^ at 866.59451 (Figure S4). Fraction 3 had two putative carotenoid-like peaks, showing similar absorption spectra (λ_max_ 450 and 475 for the first peak, and λ_max_ 452 and 477 for the second peak) (data not shown). The major MS signal MH^+^ for the first peak was at 866.58174, while it was at 865.57678 for the second peak (Figure S4). The major MS signals in fractions 2 and 3 had close *m*/*z* value and absorption spectra, which generally agreed with that of C.p.450 glucoside (MH^+^: 867). However, it should be interpreted with caution, since high-resolution mass spectrometry accepts small deviation and the reported MS data of these C_50_ carotenoids were obtained using low resolution mass spectrometry. As suggested by cluster 2, *M. antarctica* ZS314^T^ is expected to produce C50 carotenoids probably similar to C.p.450 and its glucoside derivatives, the products of *Dietzia* sp. CQ4. Considering the analysis of the gene cluster and the preliminary characterization of the pigments, the carotenoids produced by *M. antarctica* ZS314^T^ are most likely C.p.450 and its glucosylated derivatives.

Carotenoids have many biological activities and biotechnological applications, including antioxidant activity, absorbing light and energy, transporting oxygen, antitumor activity and enhancing antibody production etc. [31,32]. The most apparent activity of carotenoids is their excellent antioxidant activity, due to their electron rich polyene chains proficient in quenching reactive oxygen species [8]. The formation of iron ferrocyanide (Prussian blue) in the ferric reducing antioxidant power (FRAP) assay due to the presence of the reddish orange pigment extracts suggested antioxidant activity of the pigments, compared with the blank control showing yellow color of potassium ferricyanide solution (Figure 7D). The absorbance of iron ferrocyanide (Prussian blue) at 700 nm (A700) further supported the above observation, with A700 of 1.045 in the pigment group on average vs. A700 of 0.085 in the control group on average. The A700 was significantly elevated in the experimental group compared with the control group, with *p* value < 0.01 (Student’s t-test). Since *M. antarctica* ZS314^T^ inhabits the intertidal sediments in Antarctica, it has to deal with UV radiation, increased oxidative stress and all other challenges caused by low temperature. Therefore, the production of carotenoids contributes to survival and increases the fitness of *M. antarctica* ZS314^T^ under the extreme environmental conditions by increasing the antioxidant capability. Moreover, the carotenoids produced by *M. antarctica* ZS314^T^ may be further explored for potential applications in biotechnology. Since *M. antarctica* ZS314^T^ has few biosynthetic gene clusters and hence a relatively simple background, carotenoids are the major products in the crude extracts, as indicated by the HPLC analysis as well (Figure 7B). Therefore, it simplifies further purification and isolation of carotenoids from *M. antarctica* ZS314^T^ for industrial purposes.

## 3. Materials and Methods 

### 3.1. Strain and Culture Conditions

*M. antarctica* ZS314^T^ was isolated on Marine Agar 2216 (Difco) from intertidal sediments of the coast off the Chinese Antarctic Zhongshan Station in East Antarctica. For further morphological observation, the strain ZS314^T^ was grown on Marine Agar 2216 (Difco) and in Marine Broth 2216 (Difco) at 20 °C for 7 days. For analysis of reddish orange pigments, the strain ZS314^T^ was cultured in Erlenmeyer flasks containing one liter of Marine Broth 2216 (Difco) at 20 °C for seven days, under rigorous shaking at 200 rpm. 

### 3.2. Genome Sequencing and Data Mining of M. antarctica ZS314^T^

Genomic DNA was extracted using QIAamp^®^ DNA Mini kit (QIAGEN, Shanghai, China). The genome was sequenced by a combination of Illumina Hiseq 4000 and PacBio RSII sequencing platforms. Two genomic libraries with inserts of 500 bp and 10 kb were constructed for Illumina and PacBio sequencing, respectively. Clean data from Illumina and PacBio sequencing were assembled using SOAPdenovo software version 2 [33] and RS_HGAP assembly version 3 included in the SMRT Analysis version 2.3.0 (https://github.com/PacificBiosciences/SMRT-Analysis). Circularization of the assemblies was evaluated based on overlaps and using SSPACE-LongRead software [34]. Genes were predicted by using Glimmer version 3.0.2 [35]. Gene annotations were obtained by querying the databases of GenBank non-redundant protein [36], Swiss-Prot [37], KEGG [38], COG [39], GO [40], and carbohydrate-active enzymes database (CAZy) [41]. The presence of rRNA and tRNA was predicted by using rRNAmmer [42] and tRNAscan [43], respectively. The secondary metabolite BGCs were predicted by the antiSMASH program, using the relaxed parameters recommended by the program [44]. Circular map of the genome was drawn using the Circos package [45]. Phylogenetic trees were constructed by using the MEGA 7.0 software with bootstrap replication set at 1000 [46]. Metabolic pathway reconstruction was performed using the web-based server PathPred, following the reference pathway of biosynthesis of secondary metabolites (plants) with default parameters [24].

### 3.3. Extraction and Analysis of Reddish Orange Pigments

Five liters of liquid Marine Broth 2216 cultures were centrifuged at 7,000 rpm at 4 °C for 10 min. After centrifugation, the supernatant was almost colorless, and the cell pellets were reddish orange. The pigments were then extracted repeatedly with methanol from freeze-dried cell pellets. To avoid any chemical change caused by light, all manipulations during the extraction of the pigments were carried out in the dark and the containers were wrapped in aluminum foil. The extracts were then pooled and concentrated by rotary evaporation at 45 °C. 

The UV-visible absorption spectra scan of concentrated methanolic extracts was performed by using Epoch spectrophotometer system with the spectrum range of 330–700 nm. The extracts were analyzed by HPLC (waters e2695) using a C18 reversed-phase column (3.5 μm, 4.6 × 150 mm, waters) with a flow rate of 1 ml·min^−1^ and detection wavelength at 480 nm. The mobile phase was a gradient of 10 to 100% acetonitrile with 0.1% formic acid for 10 min followed by 100% acetonitrile with 0.1% formic acid for 30 min, with a flow rate of 1 mL·min^−1^. The extracts were isolated and purified by column chromatography with silica gel (200–300 mesh, Merck, Darmstadt, Germany). Thin-layer chromatography (TLC) was used to check the presence and purity of carotenoid-like compounds in the fractions separated by column chromatography before pooling. TLC was performed in TLC Silica gel 60F plates (Merck, Germany), and was developed with a 3:2 (v/v) solvent mixture of acetone and petroleum ether. Carotenoids were indicated by yellow and red colors on TLC plates (Figure S5). Fractions containing putative carotenoids were further purified by column chromatography with Sephadex LH-20 (Pharmacia Biotech, Uppsala, Sweden). Purified fractions were identified using an Agilent 6224 TOF LC/MS spectrometer, with C18 reversed-phase column (3 μm, 4.6 × 75 mm, YMCSep. Technol., Japan). The mobile phase was a gradient of 20 to 100% acetonitrile with 0.1% formic acid for 12 min, followed by 100% acetonitrile with 0.1% formic acid for 48 min, with a flow rate of 0.3 mL·min^−1^. The eluted compounds were monitored at 480 nm using a diode array detector (DAD) detector. 

### 3.4. Anti-Oxidation Assay of Reddish Orange Pigments

The anti-oxidation ability of the reddish orange pigments was measured via the ferric reducing antioxidant power (FRAP) assay [47]. The mixture of 50 μL of concentrated methanolic extracts and 50 μL of 1% potassium ferricyanide K_3_Fe[CN]_6_ were incubated in the dark for 20 min at 50 °C. Then, 50 μL of 10% trichloroacetic acid was added to the above mixture, and centrifuged for 10 min at 7000 rpm at 4 °C. After centrifugation, 20 μL of sterile deionized-distilled H_2_O and 20 μL of 0.1% ferric chloride were added to the supernatant, resulting in the production of iron ferrocyanide, i.e., Prussian blue. The absorbance of Prussian blue at 700 nm was measured by Epoch spectrophotometer system. Methanol was used as a blank control and was manipulated at the same time under the same conditions. Both the experimental group and the blank group were performed in triplicate. 

### 3.5. Data Accessibility

The complete genome of *M. antarctica* ZS314^T^ has been deposited in the GenBank database under the accession number CP017146.

## 4. Conclusions

*M. antarctica* ZS314^T^ represents a novel type of under-investigated Actinobacteria. The presence of the five putative secondary metabolite gene clusters suggested the genetic potential of *M. antarctica* ZS314^T^ in producing terpenes, alkylresorcinols, salinixanthin, oligosaccharides or amino acid-derived compounds. Combined with further experimental characterization and the gene cluster analysis provided evidence for the production of C.p.450-like carotenoids. In addition, a new xanthorhodopsin gene was discovered during the analysis of cluster 3. This work contributes to the investigation of natural product biosynthesis of *M. antarctica* ZS314^T^. The report of the complete genome also provides opportunities for further investigation of the novel actinobacterium in adaptation to the extreme Antarctic environment.

## Figures and Tables

**Table 1:** Five secondary metabolite biosynthetic gene clusters predicted in *M. antarctica* ZS314^T^ genome.

Cluster ID	Type	Gene Number	Most Similar Known Gene Cluster	Percentage of Similar Genes
1	T3PKS	7	Alkylresorcinol	100%
2	Terpene	7	Carotenoid	50%
3	Terpene	6	NA *	0
4	NRPS	7	NA *	0
5	Oligosaccharide	30	NA *	0

* NA indicates not available.

**Table 2:** A comparison of key genes within the carotenoid biosynthetic gene clusters based on amino acid sequence identities.

Cluster 2 *M. antarctica* ZS314^T^		*Corynebacterium glutamicum*		*Dietzia* sp. CQ4		*Leifsonia xyli*		*Agromyces mediolanus*	
Gene	Annotation	Gene	Identity	Gene	Identity	Gene	Identity	Gene	Identity
*orf*2	Geranylgeranyl pyrophosphate synthase	*crtE*	34%	*crtE*	34%	*crtE*	44%	*crtE*	43%
*orf*3	Phytoene synthase	*crtB*	46%	*crtB*	49%	*crtB*	58%	*crtB*	61%
*orf*4	Phytoene desaturase	*crtI*	52%	*crtI*	61%	*crtI*	61%	*crtI*	58%
*orf*5	Lycopene cyclase	*crtYe*	41%	*lbtA*	40%	*crtYe*	65%	*lctA*	50%
*orf*6	Lycopene cyclase	*crtYf*	38%	*lbtBC* ^1^	46%	*lctB*	35%	*lctB*	43%
*orf*7	Lycopene elongase	*crtEb*	56%	*lbtBC* ^2^	59%	*crtEb*	61%	*lctC*	64%

*lbt*BC^1^, the N-terminal region of LbtBC (1 to 134 residues) is homologous with lycopene cyclase domain. lbtBC^2^, the C-terminal region of LbtBC (135 to 432 residues) is homologous with lycopene elongase domain.

## Supplementary Materials

The following are available online at https://www.mdpi.com/1660-3397/17/7/388/s1, Figure S1: Circular genome map of *M. antarctica* ZS314^T^. From outer to inner circle: genome size, protein-encoding genes in forward orientation, protein-encoding genes in reverse orientation, ncRNA in forward orientation, ncRNA in reverse orientation, repeat sequences, GC plot and GC skew., Figure S2: Phylogenetic tree of SrsA from *M. antarctica* ZS314^T^ and close deposits in the NCBI non-redundant protein sequences (nr) database., Figure S3: Reconstructed biosynthetic pathway of the core structure of salinixanthin without the glucose ester modification from lycopene., Figure S4: Mass spectrometry of the purified fractions from the methanolic extracts of the pigments produced by *M. antarctica* ZS314^T^., Figure S5: TLC profile of carotenoid-like pigments indicated by yellow and red colors., Table S1: Gene organization of the five gene clusters predicted in the genome of *M. antarctica* ZS314^T^.
