# Supplementary material for: Biosynthetic Potential of a Novel Antarctic Actinobacterium Marisediminicola antarctica ZS314T Revealed by Genomic Data Mining and Pigment Characterization
Source: Mar Drugs. 2019 Jul 1;17(7):388. doi: 10.3390/md17070388 (PMC6669644; doi:10.3390/md17070388)
Supplement: Supplementary file 1 [file marinedrugs-17-00388-s001.pdf]

Supplementary materials for

Biosynthetic potential of a novel Antarctic actinobacterium *Marisediminicola antarctica* ZS314 revealed by genomic data mining and pigment characterization

Li Liao[a,#,*], Shiyuan Su[a,b,#], Bin Zhao[a,c], Chengqi Fan[d], Jin Zhang[a], Huirong Li[a], Bo Chen[a,*]

[a]SOA Key Laboratory for Polar Science, Polar Research Institute of China, 451 Jinqiao Road, Shanghai, 200136, China

[b]College of Marine Sciences, Shanghai Ocean University, Shanghai, 201306, China

[c]School of Biotechnology, East China University of Science and Technology, Shanghai, 200237, China

[d]Key Laboratory of East China Sea & Oceanic Fishery Resources Exploitation and Utilization, Ministry of Agriculture, East China Sea Fisheries Research Institute, Chinese Academy of Fishery Sciences, Shanghai, 200090, China

[#]These authors contributed equally to this work.

*Correspondence:

liaoli@pric.org.cn; chenbo@pric.org.cn

Polar Research Institute of China, 451 Jinqiao Road, Shanghai, 200136, China

Phone: 86-021-50385104; Fax: 86-021-58711663

Table S1. Gene organization of the five gene clusters predicted in the genome of *M. antarctica* ZS314.

Gene ID	start	end	length/bp	strand	annotation
Cluster 1					
orf1	355581	356897	1317	+	multidrug transporter MATE
orf2	356955	358199	1245	+	major facilitator superfamily MFS_1
orf3	358210	358905	696	+	hypothetical protein
orf4	359086	359775	690	+	haloacid dehalogenase
orf5	359786	360868	1083	-	monooxygenase
orf6	360865	361413	549	-	isoprenylcysteine carboxyl methyltransferase
orf7	361401	362468	1068	-	stilbene synthase
Cluster 2					
orf1	538745	539290	546	-	isopentenyl-diphosphate delta isomerase
orf2	539313	540383	1071	+	geranylgeranyl pyrophosphate synthase (CrtE)
orf3	540380	541261	882	+	phytoene synthase (CrtB)
orf4	541258	542859	1602	+	phytoene desaturase (CrtI)
orf5	542864	543217	354	+	lycopene cyclase (LbtA)
orf6	543214	543738	525	+	lycopene beta-elongase (LbtBC)
orf7	543735	544613	879	+	lycopene beta-elongase (LbtBC)
Cluster 3					
orf1	1181185	1181649	465	-	MarR family transcriptional regulator
orf2	1181819	1182655	837	+	xanthorhodopsin
orf3	1182674	1184125	1452	+	Lycopene beta and epsilon cyclase
orf4	1184106	1185218	1113	+	beta-carotene 15,15'-monooxygenase
orf5	1185436	1185690	255	+	hypothetical protein
orf6	1185687	1187189	1503	+	alanine glycine permease
Cluster 4					
orf1	1194970	1195938	969	+	metalloregulator ArsR/SmtB family transcription factor
orf2	1196022	1196432	411	+	heat-shock protein HtpX
orf3	1196435	1197103	669	+	arsenate reductase/phosphatase
orf4	1197799	1201707	3909	+	amino acid adenylation

					protein
orf5	1201713	1203038	1326	+	peptidase M1
orf6	1203035	1203682	648	-	hypothetical protein
orf7	1203679	1205013	1335	-	hypothetical protein
Cluster 5					
orf1	2518025	2519089	1065	+	oxidoreductase
orf2	2519086	2520108	1023	+	epimerase
orf3	2520108	2521259	1152	+	glutamine--scyllo-inositol aminotransferase
orf4	2521252	2521902	651	+	acetyltransferase
orf5	2521947	2523119	1173	+	glycosyltransferase, family 1
orf6	2523116	2524588	1473	+	polysaccharide biosynthesis protein
orf7	2524620	2525741	1122	+	DegT/DnrJ/EryC1/StrS aminotransferase
orf8	2525738	2526844	1107	+	glycosyltransferase
orf9	2526852	2528084	1233	+	glycosyltransferase group 1
orf10	2528081	2528737	657	+	hypothetical protein
orf11	2528734	2530671	1938	+	O-antigen ligase family protein
orf12	2530768	2531865	1098	-	glycosyltransferase family 4 protein
orf13	2531972	2533321	1350	-	glycosyltransferase family 1
orf14	2533477	2535285	1809	+	asparagine synthase
orf15	2535298	2536146	849	-	glycosyltransferase
orf16	2536143	2537042	900	-	glycosyltransferase family 2 protein
orf17	2537144	2538295	1152	-	hypothetical protein
orf18	2538518	2540257	1740	-	hypothetical protein
orf19	2540504	2541436	933	-	hypothetical protein
orf20	2541524	2542540	1017	-	hypothetical protein
orf21	2542537	2543826	1290	-	major Facilitator Superfamily protein
orf22	2543840	2544382	543	-	hypothetical protein
orf23	2544670	2546061	1392	+	hypothetical protein
orf24	2546830	2548143	1314	-	glycosyltransferase
orf25	2548140	2550344	2205	-	sucrose synthase (sucrose-UDP glucosyltransferase)/glycosyl transferase group 1
orf26	2550506	2551939	1434	-	flotillin family protein
orf27	2552134	2552751	618	+	flavin oxidoreductase
orf28	2552712	2554775	2064	+	acetyl-CoA synthetase
orf29	2554883	2557579	2697	+	HAD family hydrolase

orf30	2557599	2558570	972	-	nucleoside-diphosphate sugar epimerase

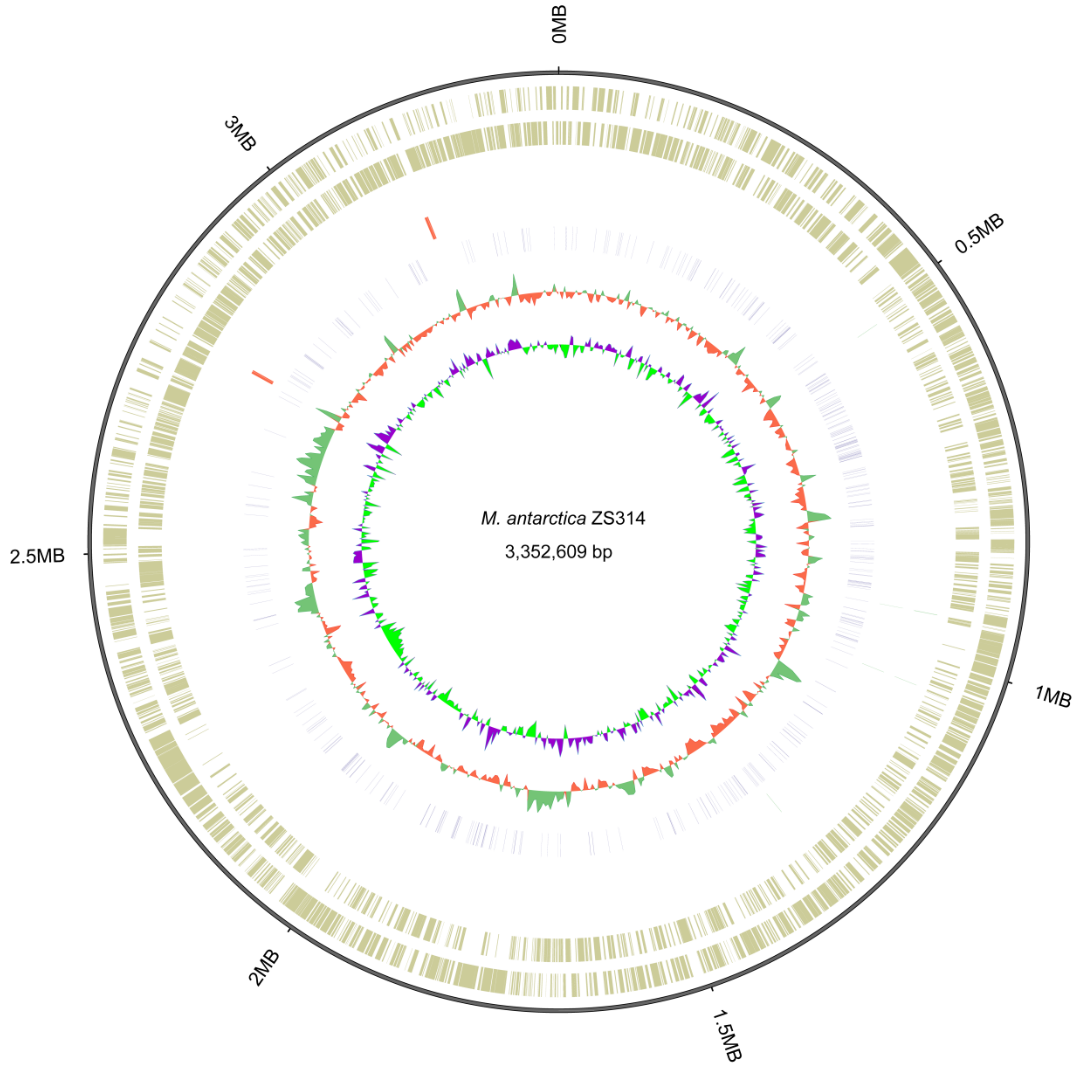

Figure S1. Circular genome map of *M. antarctica* ZS314. From outer to inner circle: genome size, protein-encoding genes in forward orientation, protein-encoding genes in reverse orientation, ncRNA in forward orientation, ncRNA in reverse orientation, repeat sequences, GC plot and GC skew.

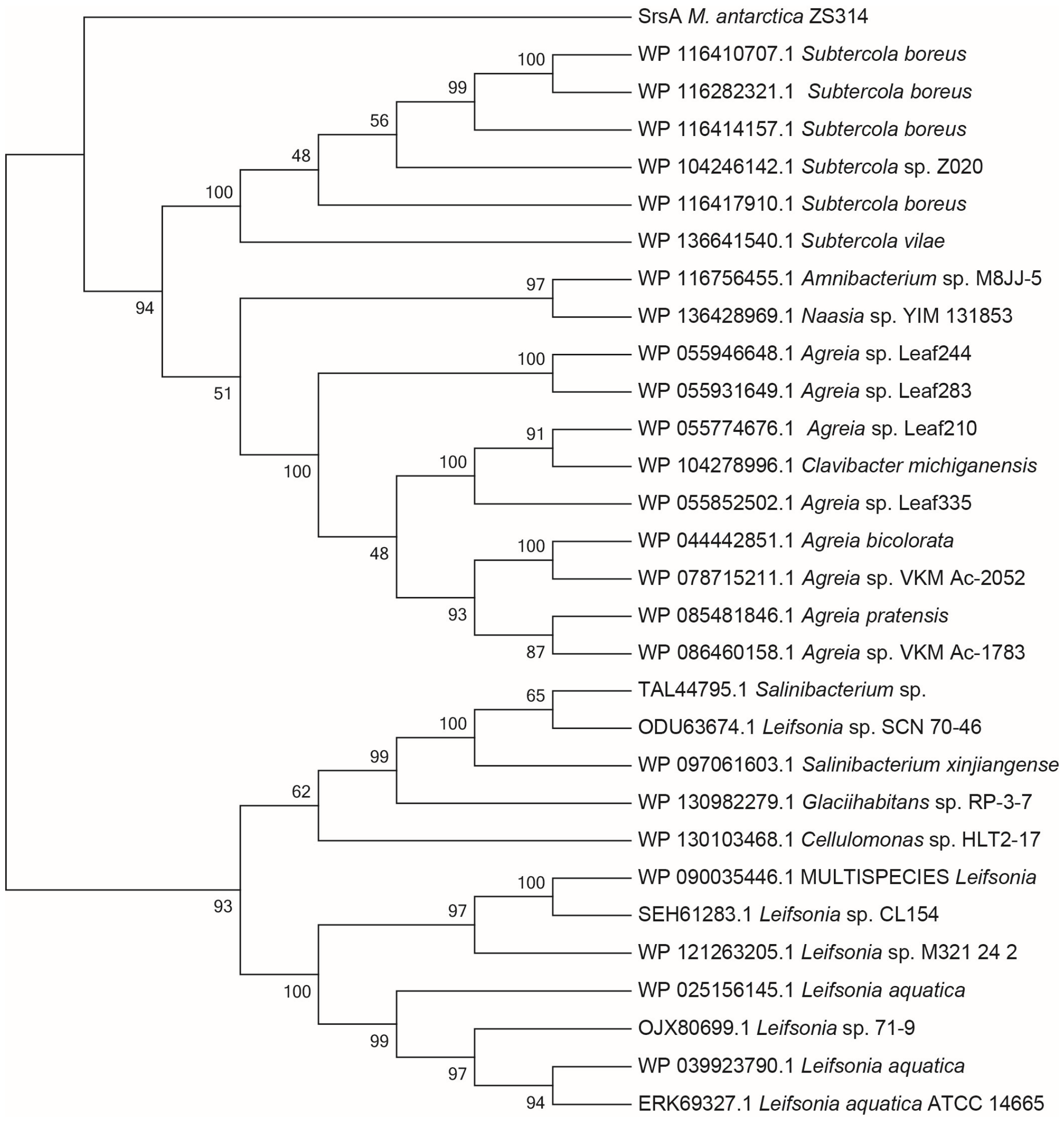

Figure S2. Phylogenetic tree of SrsA from *M. antarctica* ZS314 and close deposits in the NCBI Non-redundant protein sequences (nr) database.

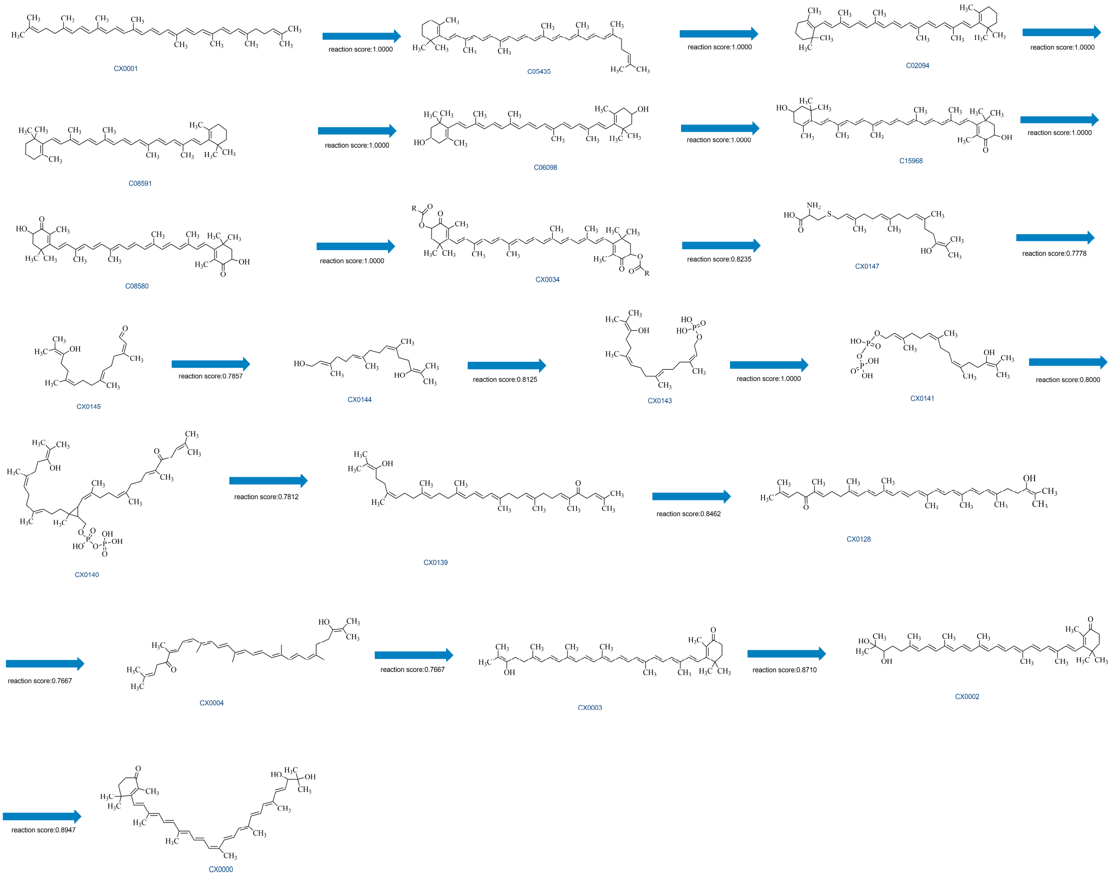

Figure S3: Reconstructed biosynthetic pathway of the core structure of salinixanthin without the glucose ester modification from lycopene.

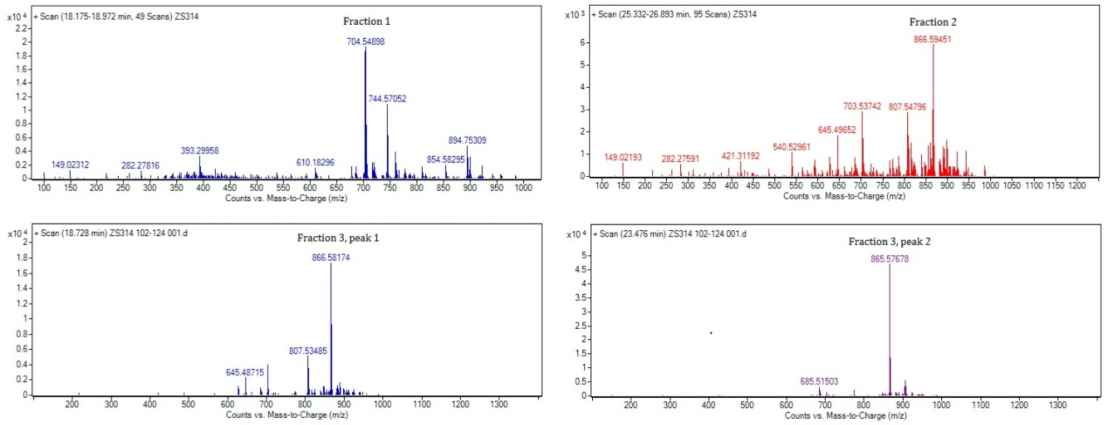

Figure S4. Mass spectrometry of the purified fractions from the methanolic extracts of the pigments produced by *M. antarctica* ZS314.

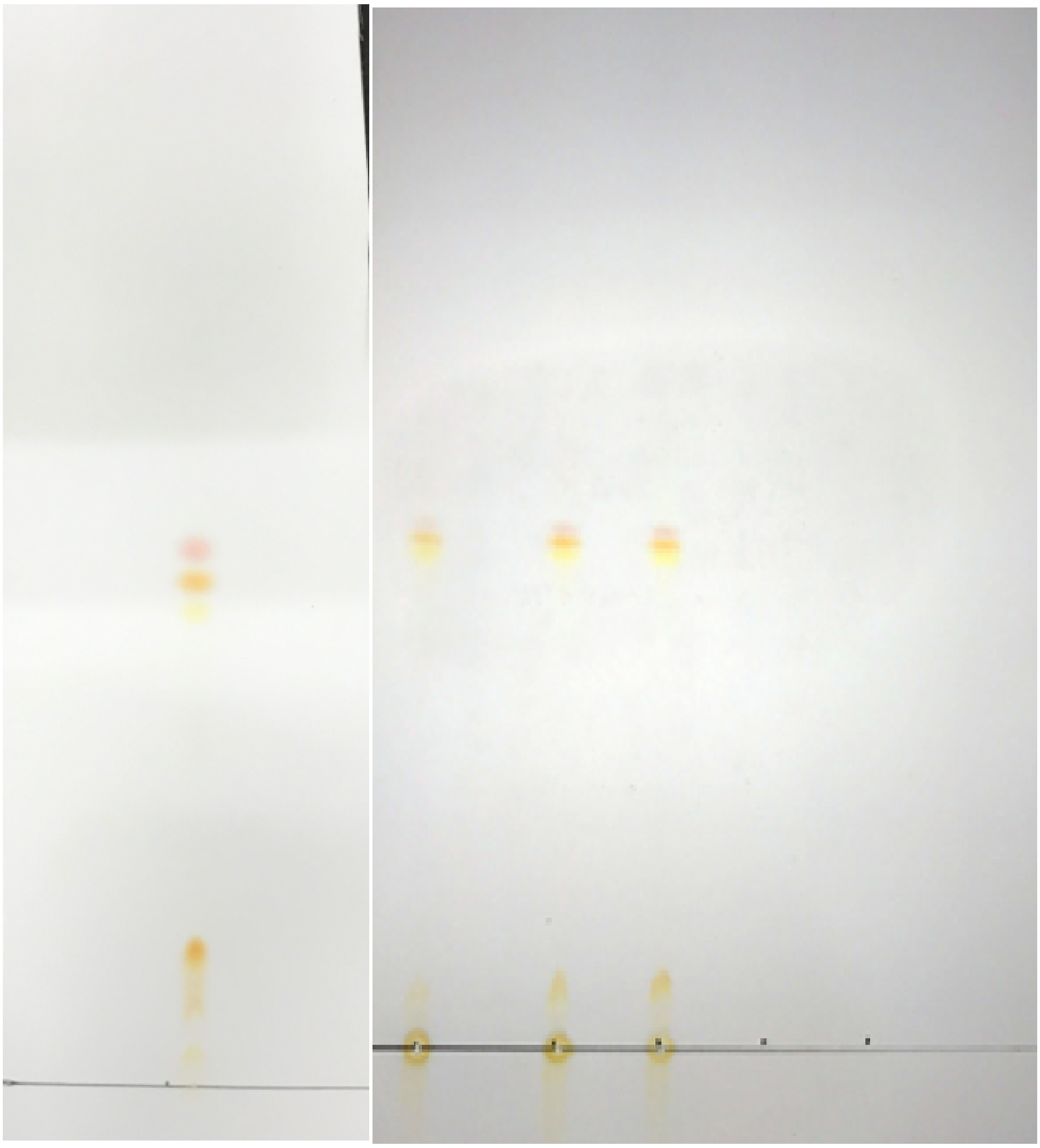

Figure S5: TLC profile of carotenoid-like pigments indicated by yellow and red colors.
